# Transcriptome Sequencing Reveals Large-Scale Changes in Axenic *Aedes aegypti* Larvae

**DOI:** 10.1371/journal.pntd.0005273

**Published:** 2017-01-06

**Authors:** Kevin J. Vogel, Luca Valzania, Kerri L. Coon, Mark R. Brown, Michael R. Strand

**Affiliations:** Department of Entomology, The University of Georgia, Athens, Georgia, United States of America; Johns Hopkins University, Bloomberg School of Public Health, UNITED STATES

## Abstract

Mosquitoes host communities of microbes in their digestive tract that consist primarily of bacteria. We previously reported that *Aedes aegypti* larvae colonized by a native community of bacteria and gnotobiotic larvae colonized by only *Escherichia coli* develop very similarly into adults, whereas axenic larvae never molt and die as first instars. In this study, we extended these findings by first comparing the growth and abundance of bacteria in conventional, gnotobiotic, and axenic larvae during the first instar. Results showed that conventional and gnotobiotic larvae exhibited no differences in growth, timing of molting, or number of bacteria in their digestive tract. Axenic larvae in contrast grew minimally and never achieved the critical size associated with molting by conventional and gnotobiotic larvae. In the second part of the study we compared patterns of gene expression in conventional, gnotobiotic and axenic larvae by conducting an RNAseq analysis of gut and nongut tissues (carcass) at 22 h post-hatching. Approximately 12% of *Ae*. *aegypti* transcripts were differentially expressed in axenic versus conventional or gnotobiotic larvae. However, this profile consisted primarily of transcripts in seven categories that included the down-regulation of select peptidases in the gut and up-regulation of several genes in the gut and carcass with roles in amino acid transport, hormonal signaling, and metabolism. Overall, our results indicate that axenic larvae exhibit alterations in gene expression consistent with defects in acquisition and assimilation of nutrients required for growth.

## Author Summary

Several mosquito species including *Aedes aegypti* transmit pathogens as adults that cause disease in humans and other vertebrates. It has also long been known that mosquitoes host bacteria in their digestive tract, which are primarily acquired during the larval stage and transstadially transmitted to adults. Our recent results indicate that axenic larvae, which lack bacteria, do not develop beyond the first instar, whereas larvae with living bacteria develop into adults. To better understand the effects of bacteria on mosquito development, we compared growth, molting and gene expression in larval *Ae*. *aegypti* that contained several species of bacteria, only one species of bacterium (*Escherichia coli*), or no bacteria. Results showed that larvae containing several species or only *E*. *coli* grew and molted very similarly while larvae with no bacteria grew minimally and never molted. A number of *Ae*. *aegypti* genes with roles in regulating growth were differentially expressed in larvae without bacteria when compared to larvae with bacteria. Overall, our results indicate that mosquito larvae without bacteria do not grow or molt because of defects in assimilating nutrients.

## Introduction

Like most animals, mosquitoes host communities of microbes in their digestive tract that consist primarily of bacteria [1–3]. Both field and laboratory studies indicate that most of these bacteria are aerobes or facultative anaerobes [3–12]. Analysis of 16S rRNA gene amplicons of select species indicates that larvae primarily contain a subset of the bacteria in their aquatic environment, while some but not all of these bacteria are present in adults [4, 7–9, 13]. In contrast, controlled experiments show that larvae contain no gut bacteria if they hatch from surface sterilized eggs and are maintained in a sterile environment [7]. Taken together, these findings indicate that mosquito larvae acquire most if not all of their microbiota from their environment and that they transstadially transmit some members of the bacterial community to adults.

*Aedes aegypti* is a key vector of several human pathogens including filarial nematodes and the viruses that cause yellow fever, Dengue fever, Zika fever and Chikungunya [14, 15]. *Ae*. *aegypti* is also an important model for many fundamental studies on mosquito development, immunity and behavior [16–18]. Larvae reared under conventional (non-sterile) conditions and fed a nutritionally complete diet molt through four instars before pupating and emerging as adults [19]. Studies dating back to the 1920s noted that *Ae*. *aegypti* and other species of mosquito larvae contain bacteria in their gut [20–23], but conclusions regarding the role of these bacteria in development vary. Some report that bacteria are a source of nutrients or provide other factors that are required for development [23, 24] while others report that larvae develop on both undefined and defined diets in the absence of bacteria [20, 25, 26]. A key challenge in interpreting these variable findings is that researchers during this period lacked the molecular tools needed to characterize the gut microbiota in mosquitoes or determine whether larvae reported to lack bacteria actually were ‘germ free’. As a result, it is also difficult to evaluate the accuracy of the findings reported.

Using high-throughput sequencing approaches, we previously determined that a laboratory population of *Ae*. *aegypti* (UGAL strain) contains ~100 bacterial operational taxonomic units (OTUs) during the larval stage with lower bacterial diversity in adults [7]. Our experiments also indicated that axenic larvae, conclusively shown to have no bacteria, die as first instars when fed a standardized diet and maintained under sterile conditions [7, 27]. Axenic larvae also die as first instars if standard diet is supplemented with dead bacteria or is preconditioned by co-culture with living bacteria before feeding. However, axenic larvae develop into adults if colonized by bacteria from water containing conventionally reared larvae [7]. Gnotobiotic *Ae*. *aegypti* larvae colonized individually by several members of the bacterial community in conventionally reared larvae or the non-community member *Escherichia coli* also develop normally with adults showing no morphological defects or reductions in fitness as measured by development time, size and fecundity [7, 27]. Lastly, offspring from field collected *Ae*. *aegypti* and several other mosquito species host communities of bacteria that differ from laboratory cultures but exhibit the same dependency on living bacteria for development as UGAL strain *Ae*. *aegypti* [28]. Altogether, we conclude from these results that several mosquito species fail to develop if reared under axenic conditions but larvae develop normally into adults if living bacteria are present in the digestive tract. Our results further indicate that development does not depend on a particular OTU or community of bacteria in the larval digestive tract.

These findings are important because they implicate gut bacteria as a key factor in the development of larvae into adults, which is the life stage that transmits vector borne pathogens to humans. Understanding the interactions between larval stage mosquitoes and gut bacteria is also important because many of the OTUs in larvae are transstadially transmitted to adults where they can affect vector competence to transmit *Plasmodium* and arboviruses (summarized by [2, 29]). In this study, we further assessed *Ae*. *aegypti* development by comparing the growth and abundance of bacteria in conventional larvae, gnotobiotic larvae colonized by only *E*. *coli* and axenic larvae during the first instar. Based on these data, we then performed a transcriptome analysis of larvae in each treatment as a first step to understanding how bacteria in the gut affect gene expression in first instars. Our results indicated that conventional and gnotobiotic first instars grow similarly, whereas axenic larvae do not attain the critical size associated with molting of conventional and gnotobiotic larvae to the second instar. Our transcriptome analysis further indicated that a number of genes with functions in nutrient acquisition, metabolism, and stress were differentially expressed in axenic larvae when compared to the conventional and gnotobiotic treatments.

## Materials and Methods

### Ethics statement

Animal care and use are described in Animal Use Protocol A2014 12-013-R1 (renewal 1/28/2016), which was approved by The University of Georgia Institutional Animal Care and Use Committee (IACUC). The UGA IACUC oversees and provides veterinary care for all campus animal care facilities and is licensed by the US Department of Agriculture (USDA) and maintains an animal welfare Assurance, in compliance with Public Health Service policy, through the NIH Office of Laboratory Animal Welfare, and registration with the USDA APHIS Animal Care, in compliance with the USDA Animal Welfare Act and Regulations, 9 CFR. IACUC personnel attend to all rodent husbandry under strict guidelines to insure careful and consistent handling. The University of Georgia’s animal use policies and operating procedures facilitate compliance with applicable federal regulations, guidance, and state laws governing animal use in research and teaching including the: 1) The Animal Welfare Act, 2) Public Health Service (PHS) Policy on the Humane Care and Use of Laboratory Animals, 3) United States Government Principles for the Utilization and Care of Vertebrate Animals Used in Testing, Research and Training, 4) Guide for the Care and Use of Laboratory Animals, 5) Guide for the Care and Use of Agricultural Animals in Research and Teaching, 6) American Veterinary Medical Association Guidelines for the Euthanasia of Animals, and 7) Applicable Georgia laws.

### Insects

UGAL *Ae*. *aegypti* were maintained as previously described by feeding larvae a standardized, nutritionally complete diet (1:1:1 rat chow: lactalbumin: torula yeast) and blood-feeding adult females on an anesthetized rat [30]. Anesthetization of rats (Sprague-Dawley strain) obtained from Charles Rivers Laboratories for mosquito blood feeding was performed and monitored by trained personnel as in Animal Use Protocol A2014 12-013-R1.

All larvae used in the study hatched from eggs that were surface sterilized using previously developed methods [7]. In brief, eggs laid 5–7 days previously were submerged in a sterile petri dish containing 70% ethanol in water for 5 min followed by transfer to a second petri dish containing a solution of 3% bleach and 0.1% ROCCAL-D (Pfizer) in sterile water for 3 min, followed by a second wash in 70% ethanol for 5 min. Surface sterilized eggs were then transferred to a new sterile petri dish and washed 3 times with 10 ml of sterile water followed by transfer to a sterile 10 cm^2^ culture flask containing 15 ml sterile water and allowed to hatch for 1 hour. Axenic larvae that hatched from eggs were transferred to culture flasks that contained 10 mg of our standard rearing diet that had been sterilized by gamma-irradiation [7]. Conventional larvae were produced by adding 1 ml of water from the general lab culture to a culture flask containing axenic larvae. Gnotobiotic larvae colonized by only *E*. *coli* were produced by adding 10^8^ CFUs from an overnight culture of the K12 strain (National BioResource Project: *E*. *coli/B*. *subtilis*, National Institute of Genetics, Shizuoka, Japan) to culture flasks containing axenic larvae.

When fed a nutritionally complete diet under controlled temperature and photoperiod, *Ae*. *aegypti* larvae molt at predictable intervals with each instar being distinguished by the width of the head capsule [19]. To distinguish key traits within the first instar we monitored the growth of conventional, gnotobiotic and axenic larvae by placing newly hatched individuals in 24 well culture plates containing sterilized diet and water. Cohorts of larvae were then observed every 2 h for behavioral and morphological characters associated with feeding, apolysis, and ecdysis. Larval length was measured from the anterior border of the head to the posterior border of the last abdominal segment, which precedes the siphon tube. We also measured the width of the head capsule and prothorax from the dorsal side at their widest point. All measures were made using a Leica stereomicroscope fitted with an ocular micrometer. Critical size, which is defined as the point within an instar when a larva achieved sufficient size to molt, was confirmed by transferring larvae from wells containing diet at specific times post-hatching to wells containing only sterile water. The number of larvae that molted to the second instar was then determined.

### Bacterial abundance and immunofluorescence microscopy

We estimated the number of bacteria in conventional, gnotobiotic and axenic first instars by two methods: colony count analysis of culturable bacteria and quantitative real time PCR (qPCR). Colony count data were generated as previously described [7] by collecting and surface sterilizing larvae at 18 h post-hatching followed by homogenization in LB broth and culturing on LB plates at 27° for 72 h. The number of bacterial colonies was then counted. For qPCR assays, an absolute standard curve was generated by PCR amplification using the universal bacterial 16S primers HDA1 (ACTCCTACGGGAGGCAGCAGT) and HDA2 (GTATTACCGCGGCTGCTGGCA) [31] and bacterial DNA from K12 *E*. *coli* as template followed by TOPO-TA cloning of the product as previously described [32]. After propagation in *E*. *coli*, plasmid was purified using the GeneJet Miniprep kit (Thermo Scientific). A standard curve was then generated by serial dilution of the plasmid (10^7^–10^2^ copies) and qPCR analysis. Bacterial DNA was then isolated from individual conventional, gnotobiotic and axenic larvae as previously described [7] followed by qPCR using the same primers and fitting the data to the standard curve to estimate bacterial abundance via amplicon copy number [32].

Digestive tracts were dissected for immunofluorescence microscopy from conventional, gnotobiotic and axenic larvae at 18 h post-hatching in phosphate buffer saline (PBS, pH 7.4). Samples were fixed in 4% paraformaldehyde in PBS for 20 min at room temperature. After rinsing three times in PBS, guts were dehydrated in ethanol, permeabilized for 20 min in PBS plus 0.2% Triton X-100 (PBT) for 20 min, and then rewashed three times in PBT. After blocking for 1 h in PBS containing 5% goat serum (Sigma) and 0.1% Tween 20 (vol/vol) (PBS-GS-T), samples were incubated overnight at 4°C with a mouse anti-peptidoglycan primary antibody (GTX39437 GeneTex) diluted 1:200 in PBS-GS-T. After washing three times for 10 min in PBS-GS-T, samples were incubated at room temperature for 2 h with an Alexa Fluor 488 goat anti-mouse secondary antibody (Thermo Fisher) diluted 1: 2000 in PBS-GS-T. After three washes in PBS, samples were incubated overnight at 4°C with a Cy3-labeled chitin binding protein [33] diluted 1:5, followed by rinsing in PBS, and mounting on slides in 50% glycerol diluted in PBS containing 1 μg/ml HOECHST 33342 (Sigma). Samples were then examined using a Zeiss LSM 710 inverted confocal microscope with acquired images processed using Adobe Photoshop CS4. Gnotobiotic larvae colonized by K12 strain *E*. *coli* that constitutively expressed green fluorescent protein (GFP) were also processed and examined as described above.

### RNA preparation for transcriptome studies

Flasks of larvae containing conventional, gnotobiotic or axenic larvae were prepared and then used to produce RNA samples for sequencing libraries. This was done by dissecting 50 larvae per biological replicate at 22 h post-hatching in sterile PBS. Larval heads were removed and the digestive tract from each larva was collected to produce a gut sample, while the remainder of each larva formed a non-gut (carcass) sample, which consisted primarily of fat body, cuticular epithelium, the nervous system, and trachea. Each gut and carcass from a given larva was transferred to an RNase-free 1.5 ml tube. Total RNA was then extracted from each sample using TRIZol (Life Technologies) according to the manufacturer’s instructions followed by two DNAase treatments using the Turbo-DNAfree kit (Life Technologies). RNA integrity was assessed on a BioAnalyzer (Agilent) using a Eukaryotic Total mRNA Nano chip.

### Library preparation, sequencing, and data analysis

Stranded, paired-end libraries (75 bp) were constructed at the University of Georgia Genomics Core Facility for each of 18 samples: three replicates per treatment (axenic, conventional, and gnotobiotic) for each tissue (gut and carcass). Each library was barcoded and equal amounts of the libraries were pooled and sequenced on an Illumina NextSeq mid-output flowcell. Resulting FASTQ sequences were de-multiplexed and quality filtered using the FASTX-toolkit (http://hannonlab.cshl.edu/fastx_toolkit/). Reads with Phred-equivalent scores of < 30 (corresponding to a per-base error rate of 0.1%) for any base were omitted from further analysis. Reads were then re-paired and mapped to the *Ae*. *aegypti* genome ([34]; assembly AaegL3, geneset AaegL3.3) using TopHat2 [35]. Read counts and differential expression were determined using the Cufflinks package [36]. This generated fragments per kilobase of transcript per million reads mapped (FPKM) values for *Ae*. *aegypti* gene expression. This analysis also identified novel transcripts not present in the L3.3 annotation of the *Ae*. *aegypti* genome [36]. Un-annotated transcripts were further analyzed using TransDecoder, which is part of the Trinity package [37] that identifies potential protein-coding genes. Gene Ontology (GO) terms were obtained from VectorBase annotations.

### Data analyses

Larval growth and bacterial colony count assays were analyzed by either one-way analysis of variance (ANOVA) followed by post-hoc Tukey-Kramer Honest Significant Difference (HSD) tests or Fisher’s Exact Test using R (http://www.r-project.org/). Pairwise analyses between treatments and tissues of transcript abundance data were performed in Cufflinks and significance cutoffs were made at a false discovery corrected p ≤ 0.05 [35].

## Results

### Conventional and gnotobiotic first instars grow similarly whereas axenic first instars exhibit reduced growth

**Fig 1:**
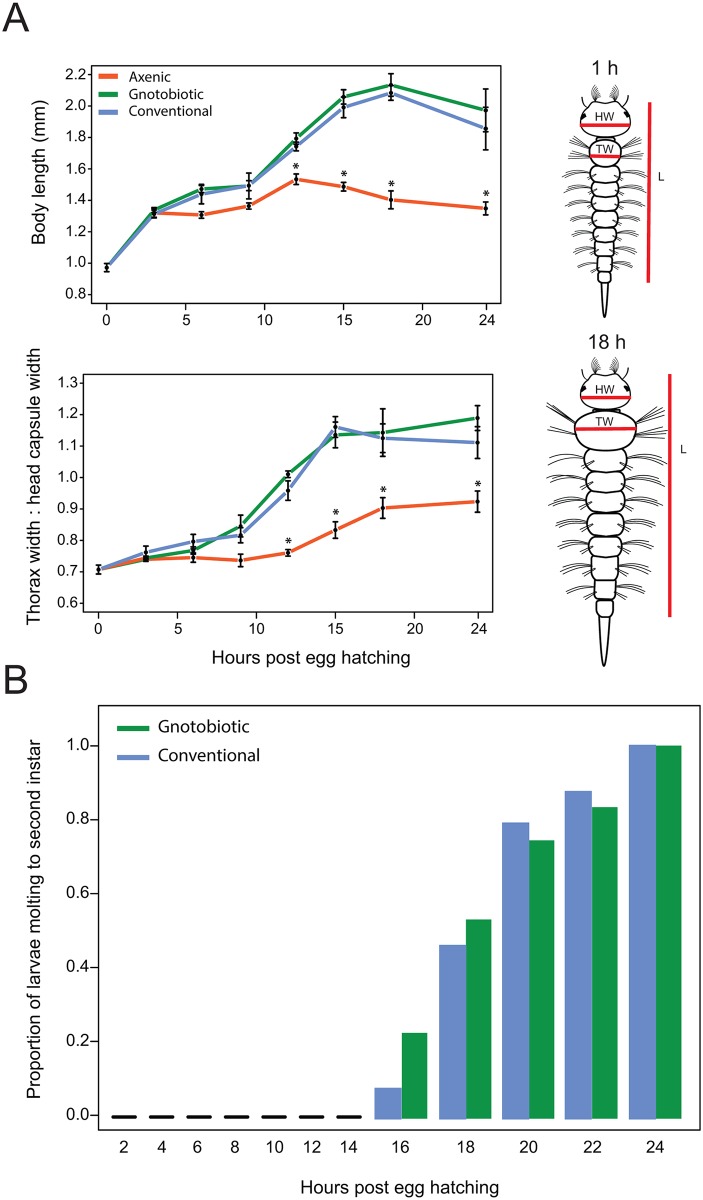
Growth of axenic, gnotobiotic and conventional *Ae*. *aegypti* as first instars. (A) Mean body length (upper graph) and the ratio of thorax width: head capsule width (lower graph) for each treatment from the time of hatching (0 h) to 24 h. A minimum of 6 individuals was measured at each time point per treatment. Each data point indicates mean value ± the standard error (SE). ANOVA analyses were conducted separately for each time point with multiple comparisons performed by Tukey-Kramer HSD test. Conventional and gnotobiotic larvae did not differ for either size measure at any time point. An asterisk (*) indicates the time points where axenic larvae significantly differ from the gnotobiotic and conventional treatments (P ≤ 0.01). To the right of each graph are drawings of 1 and 18 h post-hatching first instars showing where length (L), head capsule width (HW) and thorax width (TW) were measured. (B) Proportion of gnotobiotic and conventional larvae that molt to the second instar after transfer to wells containing water but no food. Individual larvae from each treatment were removed from culture plates containing food at two hour intervals, rinsed 3x in sterile water, and transferred to wells of a 24-well culture plate containing sterile water only. The proportion of larvae molting to the second instar was recorded at 36 h post-hatching. A minimum of 24 individuals was assayed for each treatment per time point. There were no differences between the proportion of gnotobiotic and conventional larvae that molted at any time point (Fisher’s exact test: P > 0.05).

All first instars hatched with an average head-capsule diameter of 281.7 ± 9.8 (SE) μm. Conventional and gnotobiotic larvae began feeding within 1 h of hatching (0 h) which continued for ~16 h post-hatching as evidenced by the presence of food in the gut and a corresponding increase in body size as measured by length (Fig 1A). We also noted that the width of the prothorax was less than the width of the head capsule at hatching but by 16 h was greater than the width of the head capsule (Fig 1A). These morphological features at 16 h post-hatching were associated with individuals becoming somewhat more sedentary and also not increasing further in length until after molting to the second instar (Fig 1A). Ecdysis to the second instar occurred on average at 23.5 ± 1.2 h for conventional and 23.4 ± 0.9 h for gnotobiotic larvae (t = 0.3; P > 0.1). Collectively, we interpreted these data as suggesting that conventional and gnotobiotic larvae achieved critical size and initiated apolysis at a similar time in the first instar (~16 h), which resulted in larvae from both treatments also molting to the second instar at near identical times. Experimental support for these conclusions derived from transferring conventional and gnotobiotic larvae at different times post-hatching to wells without food and assessing whether or not they could molt to the second instar. Results showed that no larvae in either treatment molted if transferred to wells without food prior to 16 h, whereas ~50% molted if transferred at 18 h, and >85% molting if transferred at 20 h (Fig 1B). Prior results showed that axenic larvae consume food [7]. However, measurements made in the current study showed that axenic first instars exhibited less growth as measured by length and the ratio of thorax: head capsule width, which remained <1 (Fig 1A). In turn, no axenic larvae ever molted, which resulted in all individuals ultimately dying as first instars.

### Conventional and gnotobiotic first instars contain similar numbers of bacteria that are similarly distributed in the gut

**Fig 2:**
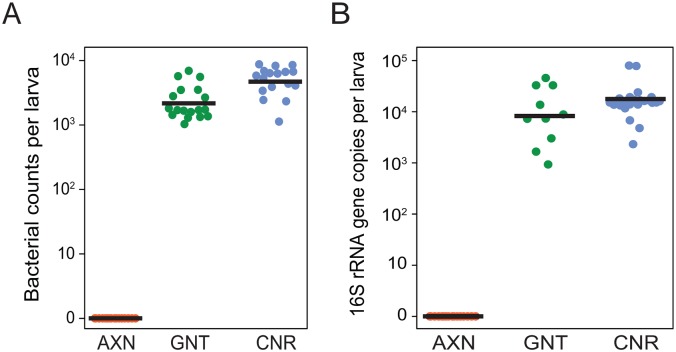
Bacterial loads in axenic (AXN), gnotobiotic (GNT) and conventional (CNR) larvae at 18 h post-hatching. Bacterial load estimated by (A) the number of bacterial colonies that grew on LB plates from homogenates of individual larvae for each treatment or (B) qPCR analysis of bacterial 16S rRNA gene copy number from individual larvae. A minimum of 17 individuals was assayed per treatment followed by ANOVA and a Tukey Kramer HSD test. Bacterial loads did not significantly differ between gnotobiotic and conventional larvae for either colony counts or qPCR-based measures, but these treatments strongly differed from axenic larvae that contained no culturable bacteria (F_2,56_ = 2527; P < 0.0001) or detectable bacterial 16S amplicons (F_2,55_ = 1292; P < 0.0001).

Previous studies indicated that conventionally reared *Ae*. *aegypti* larvae contain gram negative aerobes or facultative anaerobes that are obtained from the water where they feed [7, 28]. Several of these OTUs as well as *E*. *coli* used to colonize gnotobiotic larvae can also be cultured on Luria Broth (LB) plates at 27° [7]. We therefore used a colony count assay as a first step to estimating the number of bacteria in individual larvae at 18 h post-hatching. Results indicated that the mean number of bacteria culturable on LB plates was higher in conventional (5374.9 ± 550 (SE)) than gnotobiotic larvae (2632.6 ± 414.4) but this difference was not significant due to inter-individual variation (Fig 2A). As expected, no culturable bacteria were present in axenic larvae (Fig 2A).

Since some bacteria in conventional larvae are potentially not culturable on LB plates, we also estimated bacterial abundance using culture-independent qPCR and universal primers that amplify a conserved region of the bacterial 16S rRNA gene. 16S gene copy number did not significantly differ between conventional (19,852 ± 3,841 16S copies) and gnotobiotic (15,418 ± 3,841 16S copies) larvae, and no 16S amplicons were generated from axenic larvae (Fig 2B). However, mean values generated by qPCR were also 3.7x higher for conventional and 5.86x higher for gnotobiotic larvae than colony count estimates. This likely reflected that many bacteria encode multiple 16S operons [38, 39] and individual cells can be polyploid [40]. qPCR can also capture DNA from both living and dead bacteria. The impact of copy number is well illustrated by K12 *E*. *coli*, which is fully culturable on LB plates but contain 7 16S rRNA operons [38]. Dividing the mean 16S copy number for gnotobiotic larvae by 7 yielded a value of 2203, which was very similar to the estimate generated by colony count. We did not know 16S copy numbers for each of the OTUs in conventional larvae but the same reasoning suggested qPCR estimates were consistent with colony count data. It also suggested that the higher values generated by qPCR versus colony counts more likely reflects 16S copy number than an abundance of bacteria that were not culturable under the conditions we used.

**Fig 3:**
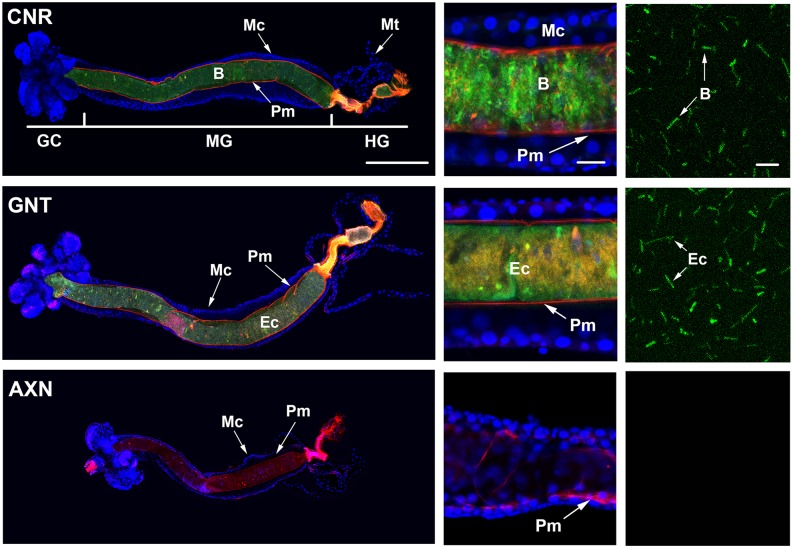
Representative images of digestive tracts from conventional (CNR), gnotobiotic (GNT) and axenic (AXN) larvae at 18 h post-hatching. The left panels show low magnification images of guts from CNR (top), GNT (middle) and AXN (bottom) larvae. The foregut was removed in each image resulting in gastric caecae (GC), midgut (MG) and hindgut (HG) being oriented from left to right. Cell nuclei were stained with Hoechst 55532 (blue) while the peritrophic matrix (Pm) was stained with a Cy3 labeled chitin binding protein (red). Midgut cell nuclei (Mc) and the Malpighian tubules are indicated. A peptidoglycan primary antibody visualized by an Alexafluor 488 secondary antibody (green) labeled bacteria (B) in the digestive tract of CNR larvae or *E*. *coli* (Ec) in gnotobiotic larvae. Note the absence of a peptidoglycan signal in AXN larvae. Scale bar in the CNR panel equals 200 μm. The middle panels show higher magnification images of the midgut for each treatment. Note that the peptidoglycan signal for bacteria (B) in the CNR treatment and *E*. *coli* (Ec) in the GNT treatment is within the endoperitrophic space formed by the Pm, whereas no signal is visible in the AXN treatment. Scale bar in the upper middle panel equals 20 μm. The right panels show high magnification images for the anti-peptidoglycan signal inside the endoperitrophic space of each treatment. This signal is predominantly associated with rod-shaped bacteria (B) in the CNR treatment and rod shaped *E*. *coli* (Ec) in the GNT treatment. No signal is detected in the AXN treatment. Scale bar in the upper panel equals 5 μm.

We examined the distribution of bacteria in the digestive tract of conventional and gnotobiotic larvae using an anti-peptidoglycan antibody, a Cy3-labeled chitin binding protein that labeled the peritrophic matrix, and Hoechst 33342 that labeled gut cell nuclei (Fig 3). In the case of gnotobiotic larvae, distribution was also visualized using *E*. *coli* that constitutively expressed GFP (S1 Fig). Results showed the presence of bacteria in the foregut, midgut and hindgut of conventional and gnotobiotic larvae (Fig 3, S1 Fig). All bacteria in the midgut also resided within the endoperitrophic space formed by the peritrophic matrix (Fig 3, S1 Fig). Anti-peptidoglycan and GFP signal intensity were similar between conventional and gnotobiotic larvae, which was consistent with our colony count and qPCR data that did not detect any differences in bacteria abundance (Fig 3, S1 Fig). Higher magnification images also clearly indicated that anti-peptidoglycan bound to particles in the endoperitrophic space that morphologically appeared to be rod-shaped bacteria (Fig 3). In contrast, anti-peptidoglycan did not detect any bacteria that were in contact with midgut cells (Fig 3). As expected, anti-peptidoglycan did not bind to any particles in the guts of axenic larvae but binding of Cy3-labeled chitin binding protein clearly showed that the midgut of axenic larvae was lined with a peritrophic matrix (Fig 3).

### Transcriptional profiling

We used Illumina sequencing to transcriptionally profile conventional, gnotobiotic and axenic first instars at 22 h post-hatching which was a time point that preceded molting of conventional and gnotobiotic first instars, whereas axenic larvae remained below critical size (see Fig 1). We also profiled the gut and carcass in each of these treatments separately. Three biological replicates per treatment and two tissue sources (gut and carcass) resulted in a total of 18 samples for which sequencing libraries were produced and analyzed. An average of 45.2 million reads were generated per sample (range: 166–10.7), which was reduced to an average of 6.3 million paired reads (range 9.9–4.4) after quality filtering (S1 Table). This resulted in a total of 15.8 to 22.9 million quality filtered reads per treatment (S1 Table) of which 67.8% on average mapped to the current assembly of the *Ae*. *aegypti* genome (AaegL3) using Tophat (S2 Table). Of the 18,293 transcripts that are annotated in the *Ae*. *aegypti* reference genome, 13,551 had an FPKM ≥1 in one or more of our samples.

**Fig 4:**
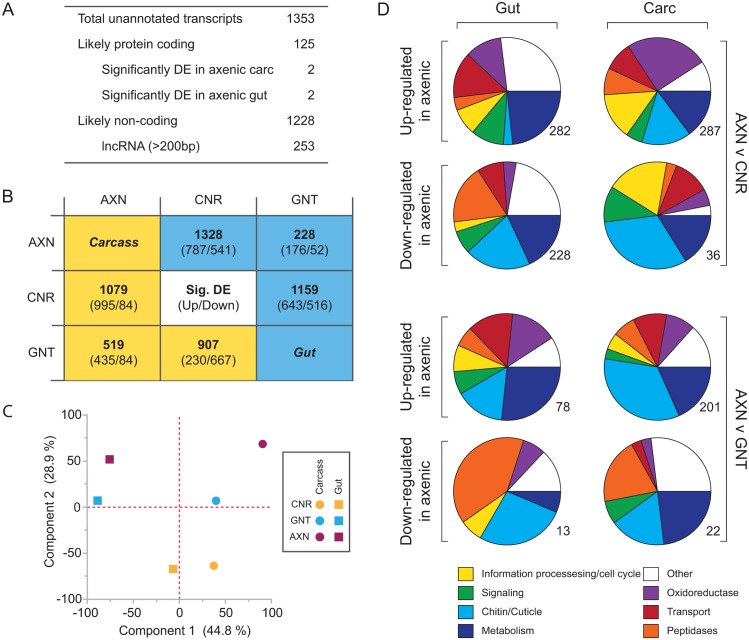
Transcriptome features in conventional, gnotobiotic and axenic *Ae*. *aegypti* first instars at 22 h post-hatching. (A) Total number of unannotated transcripts identified in Tophat that mapped to regions of the *Ae*. *aegypti* genome lacking an annotated gene. Sequences were extracted and used as input with TransDecoder to identify potential protein-coding transcripts. Remaining transcripts were analyzed with PLEK to identify potential long, non-coding RNAs (lncRNA). (B) The total number of transcripts that were significantly differentially expressed (Sig. DE) in the gut (blue) or carcass (yellow) in comparisons between axenic (AXN), conventional (CNR), and gnotobiotic (GNT) larvae. Each cell indicates a given comparison with the total number of differentially expressed transcripts indicated in bold. The numbers below this value indicate number of transcripts with mean FPKM values that were significantly higher (numerator) or lower (denominator) for the treatment indicated at the top of column when compared to the treatment shown to the left of the corresponding row. (C) Principle components analysis of transcripts that were differentially expressed in the carcass (colored circles) or gut (colored squares) in comparisons between CNR (yellow), GNT (blue), and AXN (magenta) larvae. Component 1 separates samples by tissue (carcass versus gut), while component 2 separates samples by treatment (conventional, gnotobiotic, axenic). Together, these two components accounted for 73.7% of the total variation in mean transcript FPKMs. (D) Functional clustering of the transcripts that were significantly differentially expressed between treatments in the gut or carcass (Carc). Pie charts show the GO categories to which genes that were significantly up-regulated or down-regulated in axenic versus conventional or gnotobiotic larvae belonged. GO categories with < 1% of differentially expressed transcripts in all comparisons are grouped together in the category designated as ‘Other’. The total number of transcripts assigned to functional categories is indicated beside each pie chart.

A total of 1,353 transcripts were identified that did not map to the L3 annotation of the *Ae*. *aegypti* genome (Fig 4A). Using TransDecoder, 164 of these had predicted open reading frames that were > 100 amino acids (AA), which we searched against the NCBI nr database. BLAST results detected a hit to an annotated insect gene with a bit score > 100 for 125 of these transcripts, which we interpreted as evidence they likely derive from protein coding genes that are absent from the current annotation of the *Ae*. *aegypti* genome (S2 Table). However, only 3 of these likely protein-coding transcripts were differentially expressed among treatments (Fig 4A). One of these was a conserved hypothetical protein that was more abundant in the gut and carcass of axenic versus conventional and gnotobiotic larvae. The second was a putative structural component of cuticle that was also more abundant in the carcass of axenic larvae. The third was a transcript significantly upregulated in the gut of axenic larvae that was most similar to the *Culex quinquefaciatus* gene *schnurri*: a regulatory factor in the *decapentaplegic* pathway implicated as a negative regulator of intestinal stem cell proliferation in the midgut of *D*. *melanogaster* [41]. The remaining 1,228 unannotated transcripts were presumptive non-coding RNAs of which 253 were classified using PLEK [42] as long, non-coding RNAs (Fig 4A).

To examine the number of genes that were differentially expressed between treatments, we first limited our consideration to loci with an FPKM of 10 or higher in one condition. Among the three treatments, this resulted in the number of significantly differentially expressed genes ranging from 1,328 between conventional and axenic carcasses to 228 between axenic and gnotobiotic carcasses (Fig 4B). We noted that more genes were significantly up-regulated (995) than down-regulated (84) in the carcasses of axenic larvae when compared to conventional larvae (Fig 4B). This was also the case when comparing the carcasses of axenic and gnotobiotic larvae (Fig 4B). In contrast, the number of up-regulated versus down-regulated genes was less distinctly different between the carcasses of conventional and gnotobiotic larvae or the guts of axenic, conventional, and gnotobiotic larvae (Fig 4B). Transcripts with an FPKM that was > 10 in axenic but < 1 in gnotobiotic or conventional larvae were classified as preferentially and highly up-regulated under axenic rearing conditions. Only 21 loci met these criteria with 6 being detected in the gut, 15 in the carcass, and none in both tissues. Moreover, only 3 of these loci mapped to annotated genes while 2 generated significant BLAST hits to known insect proteins. These included one acyl-CoA transferase expressed in the gut (AAEL006672) a second acyl-CoA transferase expressed in the carcass (AAEL000466), and a heat-shock 70 (HSP70) gene (AAEL017978) also expressed in the carcass. The two unannotated transcripts with significant BLAST hits were a predicted diacylglycerol kinase and an asparagine synthetase that were both expressed in the gut. The other 17 loci were unannotated with no significant BLAST hits, which suggested they were non-coding RNAs.

We further assessed large-scale differences between treatments and tissues by conducting a principle components analysis (PCA) that included all genes with an FPKM value ≥ 1 that were differentially expressed (log_2_ fold change ≥ 2) in at least one of the comparisons shown in Fig 4B (see also S3–S5 Tables). The first component, explaining 44.8% of the variation in our data, separated the samples by tissue type, which not surprisingly showed within each treatment that the differentially expressed genes identified in gut and carcass samples largely did not overlap (Fig 4C). The second component, which explained 28.9% of the variation in the data, separated the samples by treatment (Fig 4C). This indicated that the gut and carcass samples from axenic larvae most differed from conventional larvae. However, the pool of differentially expressed genes in conventional and gnotobiotic larvae also did not overlap even though larvae in both treatments grew and molted to the second instar near identically.

By extracting global classification of gene ontology (GO) terms from VectorBase, we determined that most differentially expressed genes (log_2_ fold change ≥ 2) in Fig 4B belonged to 7 functional categories: cell cycle, chitin/cuticle formation, metabolism, oxidoreductases, peptidases, signaling, and transport. Up-regulated genes in the guts and carcasses of axenic larvae were most enriched in the categories of metabolism, transport, and oxidoreductases. Most up-regulated genes in the category of oxidoreductases were cytochrome p450 enzymes (CYPs) rather than genes associated with the formation or neutralization of reactive oxygen species (S3–S5 Tables). Down-regulated genes in the guts of axenic larvae were most enriched for peptidases, while in the carcass they were most enriched for the category of chitin/cuticle (Fig 4D). Altogether, these results indicated the absence of bacteria in axenic larvae as well as the type of bacteria in conventional versus gnotobiotic larvae affected gene expression in *Ae*. *aegypti* first instars. They also indicated gene expression was affected in both gut and non-gut tissues.

### Select peptidases are down-regulated in the guts of axenic larvae while several amino acid transporters are up-regulated

**Fig 5:**
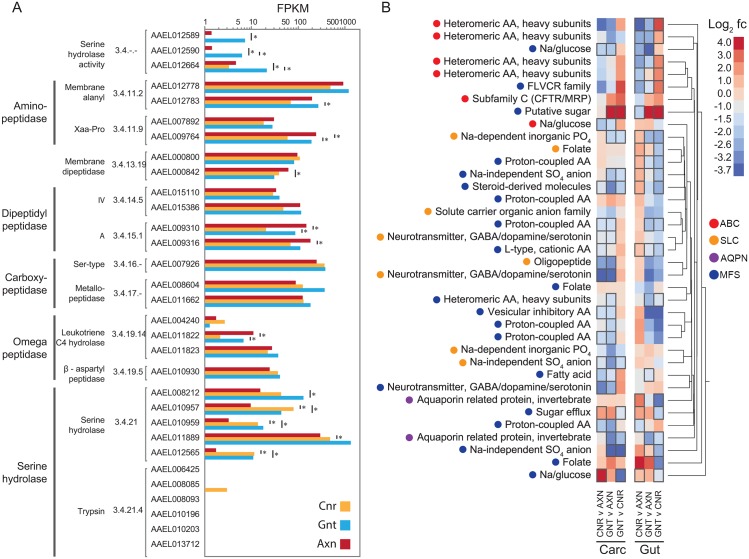
Expression of peptidase and transporter genes in the guts and carcasses of conventional (CNR), gnotobiotic (GNT) and axenic (AXN) larvae. (A) Peptidase genes with mean FPKM values > 5 in the guts of CNR, GNT or AXN larvae are presented with asterisks and underline bars indicating transcript abundances that significantly differ between particular treatments. The genes shown along the y-axis are identified by VectorBase accession and enzyme commission (E.C.) classification. At the bottom of the y axis is shown a subset of trypsin-like peptidases previously identified to be expressed in adult female *Ae*. *aegypti* and play important roles in blood meal digestion (see text). Note that most of these trypsin-like peptidases are not expressed in first instars. (B) Hierarchical clustering analysis and heatmap of expression for transmembrane transporter genes. Each column in the heatmap designates pair-wise comparisons between treatments (CNR, GNT or AXN) for either the carcass (Carc) or gut. Genes more abundantly expressed in the first condition listed at the bottom of a given column are denoted by red colors, while those more abundantly expressed in the second condition are denoted by blue colors. Color range in the heatmap indicates log_2_ fold change (fc). Gene names are listed to the left of each row while hierarchical clustering is indicated by the tree to the right of the heatmap. Colored circles denote membrane transport protein membership: ATP-binding cassette (ABC), solute carrier family (SLC), aquaporin (AQPN) and major facilitator superfamily (MFS). Black boxes surrounding entries in the heatmap indicate FPKM values that significantly differed (P ≤ 0.05) between a given pairwise treatment. Only genes showing significantly different mean FPKM values in at least one comparison are included in the heatmap.

We next focused on genes in a subset of the categories shown in Fig 4D to gain additional insights into factors that potentially contribute to the disabled growth of axenic larvae. The *Ae*. *aegypti* genome contains hundreds of peptidases but this category was of interest because of the known role peptidases play in digestion and the finding that several peptidase genes were significantly down-regulated in axenic larvae. The functional literature on digestive peptidases in *Ae*. *aegypti* is restricted to adult females where the principal enzymes identified in bloodmeal digestion are select trypsin-like serine peptidases [43–47]. However, additional trypsins or trypsin-like genes expressed in larvae have also been identified through PCR-based, expressed sequence tag (EST), or transcriptome data sets prepared from whole body samples [48–51]. The first important feature our data set revealed was that most peptidases previously identified in bloodmeal digestion were not expressed in the guts of conventional, gnotobiotic or axenic first instars (Fig 5A). Instead, several other peptidase genes exhibited FPKM values ≥50 in the gut of each treatment, while all of the peptidases with significantly lower FPKM values in axenic versus conventional and gnotobiotic larvae were serine or leukotriene-C4-hydrolases (Fig 5A). Comparing these results with another RNAseq data set [16] indicated these down-regulated peptidase genes are not expressed in the guts or carcasses of adults either before or after consumption of a blood meal. In addition, none of these genes with the exception of AAEL007926 had previously been reported to be differentially expressed in larvae [49].

The second category of interest from the perspective of digestion and nutrient acquisition was transmembrane transporters. Due potentially to lower expression of certain peptidases, several heavy subunit and proton-coupled amino acid (AA) transporter genes plus one glucose transporter had significantly higher mean FPKM values in the guts of axenic versus conventional or gnotobiotic larvae (Fig 5B). In contrast, transcript abundance of one sugar transporter was much higher in the guts of gnotobiotic than conventional or axenic larvae (Fig 5B). Several AA transporter genes as well as select neurotransmitter and sterol transporter genes were also significantly up-regulated in the carcasses of axenic larvae relative to conventional and/or gnotobiotic larvae (Fig 5B). Neurotransmitter transporters are involved in the degradation of neurotransmitters in the nervous system, and sterol transporters aid uptake and incorporation of sterols into cell and organelle membranes.

### Axenic larvae exhibit altered expression of genes with roles in growth, molting and metabolic signaling

While many genes with metabolic or signaling functions were differentially expressed between treatments, the proportion of these genes that were significantly up- or down-regulated exhibited no obvious patterns when examined by GO category distribution alone (Fig 4D). However, certain patterns did emerge when we focused on genes within these categories with essential roles in growth and molting.

**Fig 6:**
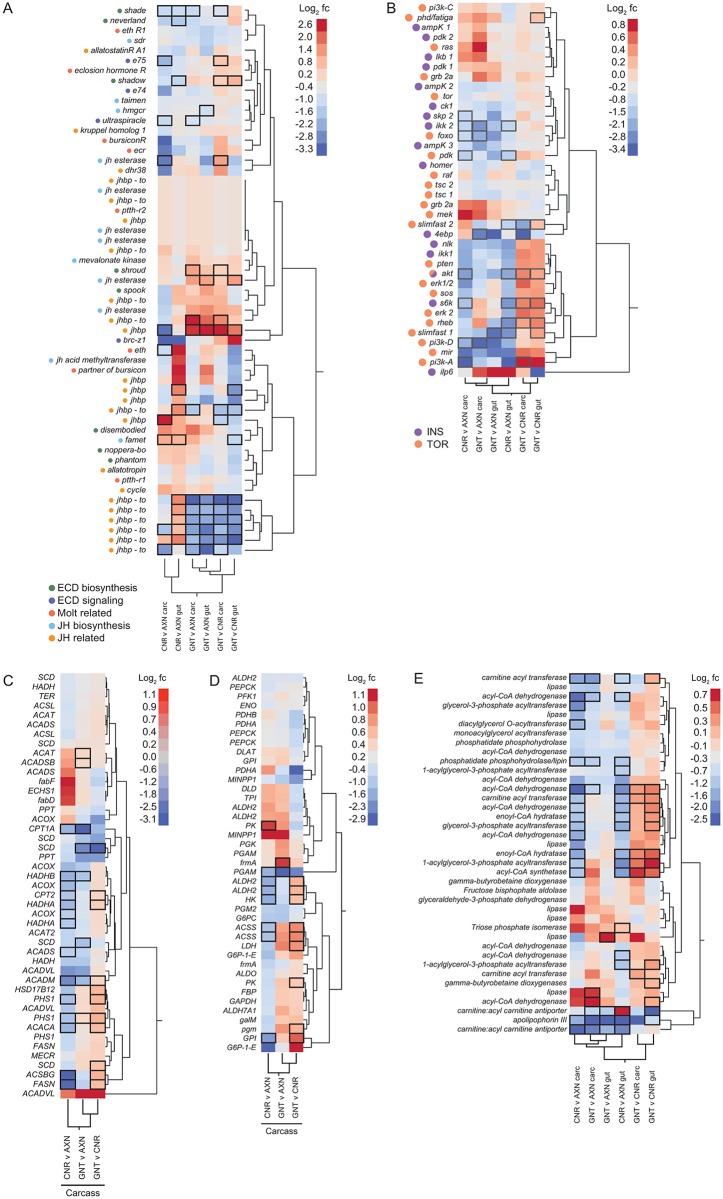
Hierarchical clustering analysis and heatmaps of expression for select groups of genes with signaling related functions. (A) Genes involved in ecdysteroid (ECD) biosynthesis (green circles), ECD signaling (dark blue circles), other molt-related activities, (orange circles), JH biosynthesis (light blue circles), and JH related activities (yellow circles). (B) Genes in the insulin signaling pathway (INS, purple circles) TOR signaling pathway (yellow circles) or both pathways (yellow/purple circles). While *Ae*. *aegypti* produces 8 insulin-like peptides (ILPs), only one is included in the heatmap because the other ILP genes are only known from expressed sequence tags (ESTs) and are not annotated. (C) Genes with functions in fatty acid metabolism. (D) Genes with functions in fatty acid β-oxidation. (E) Genes with functions in glycolysis. Labeling for each heatmap is as described in Fig 3B with gene names listed by abbreviation if well defined in the literature and VectorBase or by full spelling if not. Color range in the heatmap indicates log_2_ fold change (fc). Black boxes surrounding entries in the heatmap indicate FPKM values that significantly differed (P ≤ 0.05) between a given pairwise treatment.

The first of these gene groups that we examined focused on ecdysteroids, which regulate molting and affect larval growth [52], juvenile hormone (JH), which influences ecdysteroid function and also affects growth [53], and select other peptide hormones with roles in ecdysone and JH biosynthesis or other aspects of molting [54]. Cholesterol either stored or from the diet is converted into ecdysteroids through early steps catalyzed by *shroud*, a short-chain dehydrogenase/reductase, and *neverland*, a Rieske oxygenase, and later by the Halloween CYPs (*shadow*, *spook*, *disembodied*, *phantom*, and *shade*) [55]. Only *shroud* exhibited higher transcript abundances in the carcasses of conventional and gnotobiotic larvae when compared to axenic larvae (Fig 6A). In contrast, s*hade*, which catalyzes the conversion of ecdysone to 20-hydroxyecdysone in target tissues, was significantly more abundant in the carcasses of axenic larvae as were several downstream components of the ecdysone signaling pathway such as the ecdysteroid receptor (*ecr*), its partner *ultraspiracle*, and the downstream factor *e75* (Fig 6A). Other peptide hormones and associated receptor genes with roles in regulating ecdysone biosynthesis such as *prothracicotropic hormone* (*ptth*), or molting such as *bursicon* and *eclosion hormone*, were not differentially expressed (Fig 6A).

No significant differences were detected in mean FPKM values of *allatotropin*, *allatostatins*, or their receptors, which positively and negatively regulate JH biosynthesis in *Ae*. *aegypti* [56–58] (Fig 6A). Genes for key JH biosynthetic and metabolic enzymes including putative 3-hydroxy-3-methylglutaryl CoA reductase (*hmgr*), farnesoic acid O-methyltransferase (*famet*), and multiple predicted JH esterases also exhibited few differences among treatments (Fig 6A). In contrast, *Ae*. *aegypti* encodes multiple members of the *takeout* gene family, several of which are annotated as JH binding proteins (JHBPs) in VectorBase (*jhbp-to*) and were among the most strongly upregulated genes in the carcasses and guts of axenic larvae when compared to conventional or gnotobiotic larvae (Fig 6A). However, *takeout* genes overall share similarity with odorant binding proteins (OBPs), lipocalins and a putative JHBP (JP29) in *Manduca sexta*. Thus Takeout proteins are more broadly classified as putative hydrophobic ligand binding proteins [59]. The actual ligands for *takeout* gene family members are unknown in any insect, but studies in *Drosophila* implicate *takeout* in feeding and longevity, while also showing that starvation strongly upregulates *takeout* expression [60].

In addition to ecdysteroids and JH, growth and metabolism in insects involves the insulin signaling pathway, which converges with amino acid sensing and the target of rapamycin (TOR) pathway. FPKM values for several genes in the insulin and TOR pathways were significantly higher in the guts and carcasses of axenic versus conventional or gnotobiotic larvae (Fig 6B). Particularly striking were the increases in mean FPKM values for the insulin receptor (*mir*), *foxo*, and the FOXO target *4e-bp*, which are up-regulated in several vertebrates and invertebrates including *Ae*. *aegypti* in response to starvation or reduced nutrient availability [61–64]. No differences in expression of *mir* and *foxo* were detected when conventional and gnotobiotic larvae were compared to one another. However, select other insulin and TOR pathway genes exhibited higher mean FPKM values in gnotobiotic than conventional larvae, although fold differences were usually smaller than in comparisons between axenic and conventional or gnotobiotic larvae (Fig 6B).

Altered expression of genes in the insulin and TOR pathways in association with starvation is often coupled with up-regulated expression of genes in energy-producing metabolic pathways such as glycolysis, fatty acid metabolism, and fatty acid oxidation [61]. Mean FPKM values for several genes in each of these processes were significantly up-regulated in the guts and carcasses of axenic larvae when compared to conventional larvae (Fig 6C–6E). A lesser number of these genes were also significantly up-regulated in axenic larvae when compared to gnotobiotic larvae (Fig 6C–6E).

### Several cuticular protein genes are upregulated in axenic larvae

**Fig 7:**
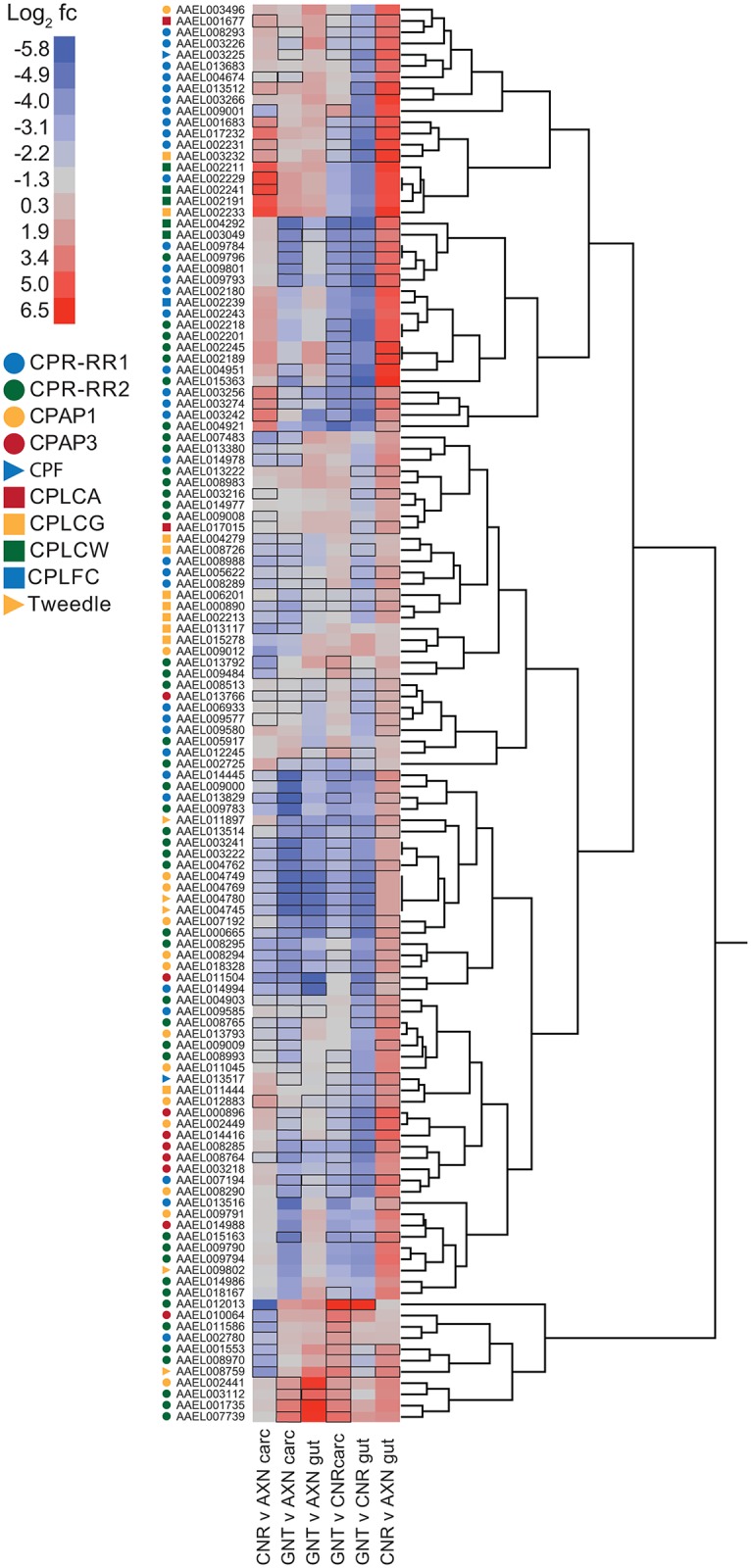
Hierarchical clustering analysis and heatmaps of expression for chitin-binding protein genes (CPs). Symbols (colored circles, squares, and triangles) to the left of the heatmap identify the ten recognized CP families. Labeling of the heatmap is as described in Fig 3B with gene name listed by VectorBase accession number and CP family assignment indicated by symbol. Color range in the heatmap indicates log_2_ fold change (fc). Black boxes surrounding entries in the heatmap indicate FPKM values that significantly differed (P ≤ 0.05) between a given pairwise treatment.

Insects including mosquitoes encode a diversity of cuticular proteins (CPs) that interact with chitin to form cuticle and/or the peritrophic matrix of the midgut [65]. A total of ten CP families are currently recognized on the basis of different motifs. These include two families distinguished by Rebers and Riddiford (RR) consensus sequences (CPR1, 2) [66], two others that are classified as Cuticular Proteins Analogous to Peritrophins (CPAP1, 3), four CP families of low complexity (CPLCA, G, W, C), and two families designated as CPF and CPT (= Tweedle) (Fig 7). Using the CP accessions curated by Ioannidou et al. [65], we determined that each had at least one member that was differentially expressed between treatments, which suggested gut bacteria broadly affect CP gene expression (Fig 7). Transcript abundance of many CP genes was significantly higher in the carcasses of axenic versus conventional and gnotobiotic larvae. However, several of the same CP genes were also differentially expressed between conventional and gnotobiotic larvae (Fig 7).

### Few immune genes are differentially expressed among treatments

Prior work establishes that bacteria in the gut induce basal level expression of genes in both the Toll and Imd pathways in adult mosquitoes [67–69] while only basal expression of the Imd pathway is induced in the digestive tract of adult *Drosophila* [70, 71]. We thus anticipated that several immune genes would likely be differentially expressed in the guts of axenic, conventional and gnotobiotic first instars. However, immune genes were not among the categories that were significantly enriched in any of our treatments (Fig 4D, S3–S5 Tables). Among the few immune genes that were differentially expressed (log_2_ fold change ≥ 2) were *pgrp-le*, which activates the Imd pathway [72, 73], and was significantly down-regulated in the guts of axenic versus conventional and gnotobiotic larvae. However, no other components of the Imd pathway were differentially expressed among treatments in either the gut or carcass (S3–S5 Tables). Three *späetzle* genes (*spz2*, *4* and *6*) which encode predicted ligands for the Toll receptor, were also down-regulated in the carcasses of axenic versus conventional larvae, but almost no other genes in or regulated by the Toll pathway, including effector proteins, were differentially expressed among treatments.

## Discussion

Our previous results indicated that several species of mosquitoes including *Ae*. *aegypti* fail to develop when fed a nutritionally complete diet and cultured under axenic conditions [7, 28]. This outcome notably contrasts with studies of *Drosophila* and mice, which show defects in maturation of the digestive tract and immune system but do not require gut microbes for development since axenic cultures of both can be maintained over multiple generations if fed a nutritionally complete diet [71, 74–77]. Only under conditions of low nutrient availability do axenic *Drosophila* larvae exhibit delays in development, which can be rescued in gnotobiotic larvae that are singly colonized by particular members of the gut community [78, 79]. Development of axenic *Ae*. *aegypti* can also be rescued in gnotobiotic larvae that are singly colonized by different species of bacteria. Unlike *Drosophila*, however, several different species of bacteria identified as community members as well as some non-community members such as *E*. *coli* rescue development of *Ae*. *aegypti* larvae, which develop at the same rate as conventionally reared larvae [7, 28]. Adult *Ae*. *aegypti* produced from gnotobiotic larvae singly colonized by *E*. *coli* also show no morphological defects or reductions in fitness when compared to adults produced from conventional larvae [27].

Altogether, these findings suggest an essential role for living microbes in development of *Ae*. *aegypti*. Axenic larvae will not develop when provided diet along with dead bacteria or diet that has been pre-conditioned by living bacteria [7]. Along with our current findings, these data argue against bacteria being an essential food source or providing a particular nutrient essential to larval development. In contrast, the absence of living bacteria in the gut could adversely affect physiological processes in larvae with roles in nutrient acquisition or assimilation. Thus, the primary goal of this study was to assess whether axenic larvae exhibit alterations consistent with this possibility or alternatively exhibit defects that point to other factors that could potentially underlie their inability to develop.

We first assessed whether conventional and gnotobiotic larvae exhibit any fine scale differences in growth during the first instar, and also whether axenic larvae exhibit specific traits that help explain why they do not molt. Our results identified no differences in growth or timing of molting between conventional and gnotobiotic first instars. The statistically similar number and distribution of bacteria in conventional and gnotobiotic larvae suggests the digestive tract of both contains sufficient space to host a finite number of bacterial cells that *E*. *coli* occupied when alone but which multiple species occupied in conventional larvae. The observation that all bacteria in conventional and gnotobiotic larvae reside inside the endoperitrophic space further suggests their essential role in growth does not involve direct contact with midgut cells. In contrast, our results indicate that axenic larvae grow a small amount but never reach the critical size associated with apolysis and other events that precede molting by conventional and gnotobiotic larvae. Studies of several insects indicate that individual species often increase in size by approximately the same factor through the penultimate instar [80, 81]. Within each instar, larvae also initiate a molt upon reaching a particular critical size, which is often associated with allometries such as the ratio between head capsule width and weight. In the first through penultimate instar, reaching critical size stimulates ecdysteroid hormone release, which induces the epidermis to produce a new cuticle while digesting most of the old endocuticle (apolysis). This is followed by ecdysis, which refers to shedding of the old exo- and epicuticle and the beginning of the next instar. In the final instar related events result in pupation. The aquatic habit and small size of *Ae*. *aegypti* first instars precluded using the ratio between head capsule width and weight to estimate when larvae achieved critical size. However, we determined that the ratio of prothorax width to head capsule width exceeds 1 when conventional and gnotobiotic *Ae*. *aegypti* first instars achieve critical size. This measure also supported the conclusion that axenic larvae do not achieve critical size.

Our transcriptome analysis at 22 h post-hatching indicated that approximately 12% of the annotated genes in the *Ae*. *aegypti* genome are differentially expressed in axenic larvae when compared to conventional or gnotobiotic larvae. However, this profile consisted primarily of genes in seven categories that included the down-regulation of select peptidases in the gut and up-regulation of several genes in the gut and carcass with roles in amino acid transport, signaling through the ecdysteroid, insulin and TOR pathways, and fatty acid oxidation. Reduced expression of select peptidases suggests the absence of bacteria may adversely affect digestion, while the increased transcription of amino acid transporters, genes associated with insulin and TOR signaling, and fatty acid oxidation suggests a response to acquire additional nutrients and use lipid reserves from embryogenesis for nourishment. Similar patterns have been observed in mammals, *Drosophila* and mosquitoes in response to starvation stress [62, 82–84]. Insulin and TOR signaling have also been implicated in affecting JH synthesis, ecdysteroid synthesis, and ecdysteroid signaling in several insects including *Ae*. *aegypti* [62, 85–89]. That *Ae*. *aegypti* encodes multiple *takeout* orthologs, which are up-regulated in axenic larvae, is also intriguing given evidence showing that *takeout* expression is strongly upregulated in *Drosophila* larvae subjected to starvation but not other stress factors [60]. As previously noted, *takeout* gene products exhibit features of OBPs, JP29, a predicted JH binding protein, and lipocalins that transport a diversity of hydrophobic molecules including retinoids, steroids, lipids and pheromones [60, 90]. The actual ligands Takeout proteins bind, however, are unknown.

A number of CP genes are differentially expressed in axenic larvae relative to conventional and gnotobiotic larvae as are several CYPs assigned to the category of oxidoreductases. The significance of these differences in regard to growth and molting are uncertain although other studies have noted the differential expression of both CPs and CYPs in response to stress factors including starvation, heat, cold, and ionizing radiation [82, 83]. Insects also continuously deposit endocuticle during the intermolt period [52], which could explain why CP transcripts are detected in both axenic larvae, which never molt, and conventional or gnotobiotic larvae that were post-critical size and in the process of molting when tissue samples were collected. In contrast, we are uncertain why so few differences were detected among our treatments in regard to expression of immune genes. At minimum our results suggest differences between first instars and prior studies conducted in adult mosquitoes [67–69]. Why such differences exist, however, will require future study.

While our primary goal was to identify differentially expressed genes in axenic larvae, our results also identified several differences between conventional and gnotobiotic larvae. This indicates that colonization of larvae by *E*. *coli* alone does not fully recapitulate gene expression patterns in conventional larvae, and that the community of bacteria in the gut affects gene activity in larvae. On the other hand the differences in gene expression detected between conventional and gnotobiotic *Ae*. *aegypti* larvae are insufficient to substantially alter growth given the similarities in when larvae molted to the second instar and recently completed results showing that conventional and gnotobiotic larvae develop into adults that exhibit no differences in size or fecundity [7, 27].

In summary, this study indicates that living bacteria in first instar *Ae*. *aegypti* affect growth and alter the expression of several genes with roles in nutrient acquisition, nutrient assimilation and stress. Since we examined only a single time point in the first instar, our transcriptome data do not identify when axenic larvae first exhibit changes in gene expression relative to conventional or gnotobiotic larvae. However, given that axenic first instars grow minimally beyond their size at hatching suggests the absence of living bacteria in the digestive tract adversely affects nutrient acquisition and/or assimilation almost immediately after hatching. We also recognize that our study did not include a treatment where conventional and gnotobiotic larvae were deprived of food to ascertain whether similar patterns are exhibited when compared to axenic larvae. We did not do this because at the onset of the investigation we did not know the key patterns our transcriptome data would identify. However, the results reported here position us to study select genes in this manner, while also providing information that will be used in functional studies of axenic larvae. In terms of disease control, the current study advances prior results by suggesting that the absence of gut bacteria disables growth at least in part by altering the metabolism of mosquito larvae and nutrient uptake. If correct, these findings further suggest that disruption of the microbial factors larvae require could potentially be used to reduce vector abundance and disease transmission [91].

## Data Deposition

Transcriptome data have been deposited in the Short Read Archive under accession PRJNA340082.

## Supporting Information

S1 Fig Representative images of bacteria in the guts of conventional (CNR) and gnotobiotic (GNT) larvae at 18 h post-hatching. Bacteria (B) in the gut of conventional larvae were labeled with a peptidoglycan primary antibody and visualized using an Alexa Fluor 488 secondary antibody (green) while *E. coli* in gnotobiotic larvae expressed green fluorescent protein. Domains corresponding to the foregut (FG), gastric caecae (GC), anterior midgut (AM), posterior midgut (PM), Malpighian tubules (MT), and hindgut (HG) are indicated at the top of the figure. Note the very similar distribution of bacteria in each treatment (scale bar = 500 μm).
(PDF)

S1 Table Quality filtering statistics of RNAseq reads. (PDF)

S2 Table Read mapping statistics. (PDF)

S3 Table Gene expression data for gut versus carcass tissues of the same treatment. Columns are (1) test_id: the unique accession of each transcript as determined by cufflinks. (2) gene: VectorBase accession(s) that map to the locus. Entries with a “-”indicate novel transcripts identified by TopHat. (3) locus: the coordinates of the *Aedes aegypti* genome assembly 3 to which the transcript mapped. (4) sample_1: the sample being compared to (5) sample_2. “axn” axenic, “cnr” conventional, “gnt” gnotobiotic, “mg” gut, “carc” carcass. (6) status: indicates whether sufficient reads were mapped to the locus to perform statistical analysis. “NOTEST” indicates sample did not have sufficient alignments to statistically test. (7) value_1 (8) value_2: FPKM values corresponding to sample_1 and sample_2, respectively. (9) log2(fold_change): the log2 fold change difference in FPKM between sample_1 and sample_2. Positive values indicate higher expression in sample_2, negative values indicate higher expression in sample_1. (10) test_stat: test statistic used to compute significance of the difference in FPKM between samples. (11) p_value: uncorrected significance of comparison in expression. (12) q_value: Benjamini-Hochberg false-discovery rate corrected p-value. (13) significant: “yes” if (12) is less than 0.05.
(TXT)

S4 Table Gene expression differences between carcass tissues from different treatments. Columns are as in S3 Table.
(TXT)

S5 Table Gene expression differences between gut tissues from different treatments. Columns are as in S3 Table.
(TXT)

## References

[1] Dennison NJ, Jupatanakul N, Dimopoulos G. The mosquito microbiota influences vector competence for human pathogens. Curr Opin Insect Sci. 2014;3:6–13. 10.1016/j.cois.2014.07.004 25584199 PMC4288011

[2] Hegde S, Rasgon JL, Hughes GL. The microbiome modulates arbovirus transmission in mosquitoes. Curr Opin Virol. 2015;15:97–102. 10.1016/j.coviro.2015.08.011 26363996 PMC5731638

[3] Minard G, Mavingui P, Moro CV. Diversity and function of bacterial microbiota in the mosquito holobiont. Parasite Vector. 2013;6:146. 10.1186/1756-3305-6-146 PMC3667145 23688194

[4] Boissiere A, Tchioffo MT, Bachar D, Abate L, Marie A, Nsango SE, et al Midgut microbiota of the malaria mosquito vector *Anopheles gambiae* and interactions with *Plasmodium falciparum* infection. PLoS Pathog. 2012;8(5): e1002742 10.1371/journal.ppat.1002742 22693451 PMC3364955

[5] Buck M, Nilsson LKJ, Brunius C, Dabire RK, Hopkins R, Terenius O. Bacterial associations reveal spatial population dynamics in *Anopheles gambiae* mosquitoes. Sci Rep. 2016;6: 22806 10.1038/srep22806 26960555 PMC4785398

[6] Chavshin AR, Oshaghi MA, Vatandoost H, Pourmand MR, Raeisi A, Enayati AA, et al Identification of bacterial microflora in the midgut of the larvae and adult of wild caught *Anopheles stephensi*: A step toward finding suitable paratransgenesis candidates. Acta Trop. 2012;121(2):129–34. 10.1016/j.actatropica.2011.10.015 22074685

[7] Coon KL, Vogel KJ, Brown MR, Strand MR. Mosquitoes rely on their gut microbiota for development. Mol Ecol. 2014;23(11):2727–39. 10.1111/mec.12771 24766707 PMC4083365

[8] Duguma D, Hall MW, Rugman-Jones P, Stouthamer R, Terenius O, Neufeld JD, et al Developmental succession of the microbiome of *Culex* mosquitoes. BMC Microbiol. 2015;15:140 10.1186/s12866-015-0475-8 26205080 PMC4513620

[9] Gimonneau G, Tchioffo MT, Abate L, Boissiere A, Awono-Ambene PH, Nsango SE, et al Composition of *Anopheles coluzzii* and *Anopheles gambiae* microbiota from larval to adult stages. Infect Genet Evol. 2014;28:715–24. 10.1016/j.meegid.2014.09.029 25283802

[10] Muturi EJ, Kim CH, Bara J, Bach EM, Siddappaji MH. *Culex pipiens* and *Culex restuans* mosquitoes harbor distinct microbiota dominated by few bacterial taxa. Parasite Vector. 2016;9:18. 10.1186/s13071-016-1299-6 PMC4712599 26762514

[11] Osei-Poku J, Mbogo CM, Palmer WJ, Jiggins FM. Deep sequencing reveals extensive variation in the gut microbiota of wild mosquitoes from Kenya. Mol Ecol. 2012;21(20):5138–50. 10.1111/j.1365-294X.2012.05759.x 22988916

[12] Yadav KK, Bora A, Datta S, Chandel K, Gogoi HK, Prasad GBKS, et al Molecular characterization of midgut microbiota of *Aedes albopictus* and *Aedes aegypti* from Arunachal Pradesh, India. Parasite Vector. 2015;8:641. 10.1186/s13071-015-1252-0 PMC4683861 26684012

[13] Wang Y, Gilbreath TM, Kukutla P, Yan G, Xu J. Dynamic gut microbiome across life history of the malaria mosquito *Anopheles gambiae* in Kenya. PLoS One. 2011;6(9):e24767 10.1371/journal.pone.0024767 21957459 PMC3177825

[14] Barrett ADT, Higgs S. Yellow Fever: a disease that has yet to be conquered. Annu Rev Entomol. 2006;52(1):209–29. 10.1146/annurev.ento.52.110405.091454 16913829

[15] Dutra HLC, Rocha MN, Dias FBS, Mansur SB, Caragata EP, Moreira LA. *Wolbachia* blocks currently circulating Zika virus isolates in Brazilian *Aedes aegypti* mosquitoes. Cell Host & Microbe. 2016;19(6):771–4. 27156023 10.1016/j.chom.2016.04.021 PMC4906366

[16] Akbari OS, Antoshechkin I, Amrhein H, Williams B, Diloreto R, Sandler J, et al The developmental transcriptome of the mosquito *Aedes aegypti*, an invasive species and major arbovirus vector. G3-Genes Genom Genet. 2013;3(9):1493–509. 10.1534/g3.113.006742 PMC3755910 23833213

[17] Attardo GM, Hansen IA, Raikhel AS. Nutritional regulation of vitellogenesis in mosquitoes: Implications for anautogeny. Insect Biochem Mol Biol. 2005;35(7):661–75. 10.1016/j.ibmb.2005.02.013 15894184

[18] Severson DW, Behura SK. Mosquito genomics: progress and challenges. Annu Rev Entomol. 2012;57:143–66. 10.1146/annurev-ento-120710-100651 21942845

[19] Timmermann SE, Briegel H. Larval growth and biosynthesis of reserves in mosquitoes. J Insect Physiol. 1999;45(5):461–70. 12770329 10.1016/s0022-1910(98)00147-4

[20] Barber MA. The food of anophiline larvae-food organisms in pure culture. US Pub Health Rep. 1927;43:11–7.

[21] Chao J, Wistreich GA. Microorganisms from the mid-gut of larval and adult *Culex quinquefasciatus*. J Insect Path. 1960;2:220–4.

[22] Ferguson MJ, Micks DW. Microorganisms associated with mosquitoes. 1. Bacteria isolated from mid-gut of adult *Culex fatigans Wiedemann*. J Insect Path. 1961;3(2):112–9.

[23] Hinman EH. A study of the food of mosquito larvae (Culicidae). Am J Hyg. 1930;12(1):238–70.

[24] Rozeboom LE. The relation of bacteria and bacterial filtrates to the development of mosquito larvae. Am J Hyg. 1935;21(1):167–79.

[25] Jones WL, Delong DM. A simplified technique for sterilizing surface of *Aedes aegypti* eggs. J Econ Entomol. 1961;54(4):813–4.

[26] Lang CA, Storey RS, Basch KJ. Growth, composition and longevity of axenic mosquito. J Nutr. 1972;102(8):1057–66. 5051860 10.1093/jn/102.8.1057

[27] Coon KL, Brown MR, Strand MR. Gut bacteria differentially affect egg production in the anautogenous mosquito *Aedes aegypti* and facultatively autogenous mosquito *Aedes atropalpus* (Diptera: Culicidae). Parasite Vector. 2016;9:375. 10.1186/s13071-016-1660-9 PMC4929711 27363842

[28] Coon KL, Brown MR, Strand MR. Mosquitoes host communities of bacteria that are essential for development but vary greatly between local habitats. Mol Ecol. 2016;25(22):5806–26. 10.1111/mec.13877 27718295 PMC5118126

[29] van Tol S, Dimopoulos G. Chapter Nine: Influences of the mosquito microbiota on vector competence. In: Raihkel AS, editor. Advances in insect physiology. 2016;51:243–91.

[30] Gulia-Nuss M, Elliot A, Brown MR, Strand MR. Multiple factors contribute to anautogenous reproduction by the mosquito *Aedes aegypti*. J Insect Physiol. 2015;82:8–16. 10.1016/j.jinsphys.2015.08.001 26255841 PMC4630150

[31] Walter J, Tannock GW, Tilsala-Timisjarvi A, Rodtong S, Loach DM, Munro K, et al Detection and identification of gastrointestinal *Lactobacillus* species by using denaturing gradient gel electrophoresis and species-specific PCR primers. Appl Environ Microb. 2000;66(1):297–303. 10.1128/aem.66.1.297-303.2000 PMC91821 10618239

[32] Burke GR, Thomas SA, Eum JH, Strand MR. Mutualistic polydnaviruses share essential replication gene functions with pathogenic ancestors. PLoS Pathog. 2013;9(5): e1003348 10.1371/journal.ppat.1003348 23671417 PMC3649998

[33] Kelkenberg M, Odman-Naresh J, Muthukrishnan S, Merzendorfer H. Chitin is a necessary component to maintain the barrier function of the peritrophic matrix in the insect midgut. Insect Biochem Mol Biol. 2015;56:21–8. 10.1016/j.ibmb.2014.11.005 25449129

[34] Nene V, Wortman JR, Lawson D, Haas B, Kodira C, Tu ZJ, et al Genome sequence of *Aedes aegypti*, a major arbovirus vector. Science. 2007;316(5832):1718–23. 10.1126/science.1138878 17510324 PMC2868357

[35] Kim D, Pertea G, Trapnell C, Pimentel H, Kelley R, Salzberg SL. TopHat2: accurate alignment of transcriptomes in the presence of insertions, deletions and gene fusions. Genome Biol. 2013;14(4):R36 10.1186/gb-2013-14-4-r36 23618408 PMC4053844

[36] Trapnell C, Roberts A, Goff L, Pertea G, Kim D, Kelley DR, et al Differential gene and transcript expression analysis of RNA-seq experiments with TopHat and Cufflinks. Nat Protoc. 2012;7(3):562–78. 10.1038/nprot.2012.016 22383036 PMC3334321

[37] Haas BJ, Papanicolaou A, Yassour M, Grabherr M, Blood PD, Bowden J, et al De novo transcript sequence reconstruction from RNA-seq using the Trinity platform for reference generation and analysis. Nature Protoc. 2013;8(8):1494–512. 23845962 10.1038/nprot.2013.084 PMC3875132

[38] Klappenbach JA, Dunbar JM, Schmidt TM. rRNA operon copy number reflects ecological strategies of bacteria. Appl Environ Microb. 2000;66(4):1328–33. 10.1128/aem.66.4.1328-1333.2000 PMC91988 10742207

[39] Stevenson BS, Schmidt TM. Life history implications of rRNA gene copy number in *Escherichia coli*. Appl Environ Microb. 2004;70(11):6670–7. 10.1128/AEM.70.11.6670-6677.2004 PMC525164 15528533

[40] Oliveira JHM, Goncalves RLS, Lara FA, Dias FA, Gandara ACP, Menna-Barreto RFS, et al Blood meal-derived heme decreases ROS levels in the midgut of *Aedes aegypti* and allows proliferation of intestinal microbiota. PLoS Pathog. 2011;7(3):qe1001320. 10.1371/journal.ppat.1001320 PMC3060171 21445237

[41] Zeng X, Han L, Singh SR, Liu H, Neumuller RA, Yan D, et al Genome-wide RNAi screen identifies networks involved in intestinal stem cell regulation in *Drosophila*. Cell Rep. 2015;10(7):1226–38. 10.1016/j.celrep.2015.01.051 25704823 PMC4420031

[42] Li AM, Zhang JY, Zhou ZY. PLEK: a tool for predicting long non-coding RNAs and messenger RNAs based on an improved k-mer scheme. BMC Bioinformatics. 2014;15:311 10.1186/1471-2105-15-311 25239089 PMC4177586

[43] Brackney DE, Isoe J, Black WC, Zamora J, Foy BD, Miesfeld RL, et al Expression profiling and comparative analyses of seven midgut serine proteases from the yellow fever mosquito, *Aedes aegypti*. J Insect Physiol. 2010;56(7):736–44. 10.1016/j.jinsphys.2010.01.003 20100490 PMC2878907

[44] Isoe J, Rascon AA, Kunz S, Miesfeld RL. Molecular genetic analysis of midgut serine proteases in *Aedes aegypti* mosquitoes. Insect Biochem Mol Biol. 2009;39(12):903–12. 10.1016/j.ibmb.2009.10.008 19883761 PMC2818436

[45] Noriega FG, Pennington JE, Barillas-Mury C, Wang XY, Wells MA. *Aedes aegypti* midgut early trypsin is post-transcriptionally regulated by blood feeding. Insect Mol Biol. 1996;5(1):25–9. 8630532 10.1111/j.1365-2583.1996.tb00037.x

[46] Noriega FG, Wang XY, Pennington JE, Barillas-Mury CV, Wells MA. Early trypsin, a female-specific midgut protease in *Aedes aegypti*: Isolation, amino-terminal sequence determination, and cloning and sequencing of the gene. Insect Biochem Mol Biol. 1996;26(2):119–26. 8882654 10.1016/0965-1748(95)00068-2

[47] Noriega FG, Wells MA. A molecular view of trypsin synthesis in the midgut of *Aedes aegypti*. J Insect Physiol. 1999;45(7):613–20. 12770346 10.1016/s0022-1910(99)00052-9

[48] Despres L, Stalinski R, Faucon F, Navratil V, Viari A, Paris M, et al Chemical and biological insecticides select distinct gene expression patterns in *Aedes aegypti* mosquito. Biol Lett. 2014;10(12):20140716 10.1098/rsbl.2014.0716 25540155 PMC4298186

[49] Paris M, Melodelima C, Coissac E, Tetreau G, Reynaud S, David JP, et al Transcription profiling of resistance to Bti toxins in the mosquito *Aedes aegypti* using next-generation sequencing. J Invertebr Path. 2012;109(2):201–8. 22115744 10.1016/j.jip.2011.11.004

[50] Soares TS, Watanabe RM, Lemos FJ, Tanaka AS. Molecular characterization of genes encoding trypsin-like enzymes from *Aedes aegypti* larvae and identification of digestive enzymes. Gene. 2011;489(2):70–5. 10.1016/j.gene.2011.08.018 21914468

[51] Venancio TM, Cristofoletti PT, Ferreira C, Verjovski-Almeida S, Terra WR. The *Aedes aegypti* larval transcriptome: a comparative perspective with emphasis on trypsins and the domain structure of peritrophins. Insect Mol Biol. 2009;18(1):33–44. 10.1111/j.1365-2583.2008.00845.x 19054160

[52] Nijhout HF, Riddiford LM, Mirth C, Shingleton AW, Suzuki Y, Callier V. The developmental control of size in insects. Wiley Interdiscip Rev Dev Biol. 2014;3(1):113–34. 10.1002/wdev.124 24902837 PMC4048863

[53] Jindra M, Palli SR, Riddiford LM. The juvenile hormone signaling pathway in insect development. Annu Rev Entomol. 2013;58(1):181–204. 22994547 10.1146/annurev-ento-120811-153700

[54] Strand MR, Brown MR, Vogel KJ. Chapter six—Mosquito peptide hormones: diversity, production, and function. In: Raihkel AS, editor. Advances in insect physiology. Academic Press; 2016;51:145–88. 10.1016/bs.aiip.2016.05.003 PMC6338476 30662099

[55] Gilbert LI. Halloween genes encode P450 enzymes that mediate steroid hormone biosynthesis in *Drosophila melanogaster*. Mol Cell Endocrinol. 2004;215(1–2):1–10. 10.1016/j.mce.2003.11.003 15026169

[56] Hernández-Martínez S, Mayoral JG, Li Y, Noriega FG. Role of juvenile hormone and allatotropin on nutrient allocation, ovarian development and survivorship in mosquitoes. J Insect Physiol. 2007;53(3):230–4. 10.1016/j.jinsphys.2006.08.009 17070832 PMC2647715

[57] Li YP, Hernández-Martínez S, Noriega FG. Inhibition of juvenile hormone biosynthesis in mosquitoes: effect of allatostatic head factors, PISCF- and YXFGL-amide-allatostatins. Regul Peptides. 2004;118(3):175–82. 10.1016/j.regpep.2003.12.004 15003834

[58] Nouzova M, Brockhoff A, Mayoral JG, Goodwin M, Meyerhof W, Noriega FG. Functional characterization of an allatotropin receptor expressed in the corpora allata of mosquitoes. Peptides. 2012;34(1):201–8. 10.1016/j.peptides.2011.07.025 21839791 PMC3233642

[59] Schwinghammer MA, Zhou X, Kambhampati S, Bennett GW, Scharf ME. A novel gene from the takeout family involved in termite trail-following behavior. Gene. 2011;474(1–2):12–21. 10.1016/j.gene.2010.11.012 21134424

[60] Sarov-Blat L, So WV, Liu L, Rosbash M. The *Drosophila takeout* gene is a novel molecular link between circadian rhythms and feeding behavior. Cell. 2000;101(6):647–56. 10892651 10.1016/s0092-8674(00)80876-4

[61] Baker KD, Thummel CS. Diabetic larvae and obese flies-emerging studies of metabolism in *Drosophila*. Cell Metab. 2007;6(4):257–66. 10.1016/j.cmet.2007.09.002 17908555 PMC2231808

[62] Perez-Hedo M, Rivera-Perez C, Noriega FG. Starvation increases insulin sensitivity and reduces juvenile hormone synthesis in mosquitoes. PLoS One. 2014;9(1):e86183 10.1371/journal.pone.0086183 24489697 PMC3906049

[63] Puig O, Tjian R. Nutrient availability and growth: regulation of insulin signaling by dFOXO/FOXO1. Cell Cycle. 2006;5(5):503–5. 10.4161/cc.5.5.2501 16552183

[64] Roy SG, Raikhel AS. Nutritional and hormonal regulation of the TOR effector 4E-binding protein (4E-BP) in the mosquito *Aedes aegypti*. FASEB J. 2012;26(3):1334–42. 10.1096/fj.11-189969 22159149 PMC3289498

[65] Ioannidou ZS, Theodoropoulou MC, Papandreou NC, Willis JH, Hamodrakas SJ. CutProtFam-Pred: Detection and classification of putative structural cuticular proteins from sequence alone, based on profile Hidden Markov Models. Insect Biochem Mol Biol. 2014;52:51–9. 10.1016/j.ibmb.2014.06.004 24978609 PMC4143468

[66] Rebers JE, Riddiford LM. Structure and expression of a *Manduca sexta* larval cuticle gene homologous to *Drosophila* cuticle genes. J Mol Biol. 1988;203(2):411–23. 2462055 10.1016/0022-2836(88)90009-5

[67] Dong YM, Manfredini F, Dimopoulos G. Implication of the mosquito midgut microbiota in the defense against malaria parasites. PLoS Pathog. 2009;5(5):e1000423 10.1371/journal.ppat.1000423 19424427 PMC2673032

[68] Meister S, Agianian B, Turlure F, Relogio A, Morlais I, Kafatos FC, et al *Anopheles gambiae* PGRPLC-mediated defense against bacteria modulates infections with malaria parasites. PLoS Pathog. 2009;5(8):e1000542 10.1371/journal.ppat.1000542 19662170 PMC2715215

[69] Xi Z, Ramirez JL, Dimopoulos G. The *Aedes aegypti* toll pathway controls Dengue virus infection. PLoS Pathog. 2008;4(7):e1000098 10.1371/journal.ppat.1000098 18604274 PMC2435278

[70] Broderick NA, Buchon N, Lemaitre B. Microbiota-induced changes in *Drosophila melanogaster* host gene expression and gut morphology. mBio. 2014;5(3):e01117–14. 10.1128/mBio.01117-14 24865556 PMC4045073

[71] Buchon N, Osman D, David FP, Fang HY, Boquete JP, Deplancke B, et al Morphological and molecular characterization of adult midgut compartmentalization in *Drosophila*. Cell Rep. 2013;3(5):1725–38. 10.1016/j.celrep.2013.04.001 23643535

[72] Dziarski R. Peptidoglycan recognition proteins (PGRPs). Mol Immunol. 2004;40(12):877–86. 14698226 10.1016/j.molimm.2003.10.011

[73] Takehana A, Katsuyama T, Yano T, Oshima Y, Takada H, Aigaki T, et al Overexpression of a pattern-recognition receptor, peptidoglycan-recognition protein-LE, activates imd/relish-mediated antibacterial defense and the prophenoloxidase cascade in *Drosophila* larvae. Proc Natl Acad Sci U S A. 2002;99(21):13705–10. 10.1073/pnas.212301199 12359879 PMC129750

[74] Blaser MJ. Antibiotic use and its consequences for the normal microbiome. Science. 2016;352(6285):544–5. 10.1126/science.aad9358 27126037 PMC4939477

[75] Goodrich JK, Davenport ER, Waters JL, Clark AG, Ley RE. Cross-species comparisons of host genetic associations with the microbiome. Science. 2016;352(6285):532–5. 10.1126/science.aad9379 27126034 PMC5116907

[76] Jiang H, Lkhagva A, Daubnerová I, Chae HS, Simo L, Jung SH, et al Natalisin, a tachykinin-like signaling system, regulates sexual activity and fecundity in insects. Proc Natl Acad Sci U S A. 2013;110(37):E3526–34. 10.1073/pnas.1310676110 23980168 PMC3773796

[77] Sommer F, Backhed F. The gut microbiota—masters of host development and physiology. Nat Rev Microbiol. 2013;11(4):227–38. 10.1038/nrmicro2974 23435359

[78] Shin SC, Kim SH, You H, Kim B, Kim AC, Lee KA, et al *Drosophila* microbiome modulates host developmental and metabolic homeostasis via insulin signaling. Science. 2011;334(6056):670–4. 10.1126/science.1212782 22053049

[79] Storelli G, Defaye A, Erkosar B, Hols P, Royet J, Leulier F. *Lactobacillus plantarum* promotes *Drosophila* systemic growth by modulating hormonal signals through TOR-dependent nutrient sensing. Cell Metab. 2011;14(3):403–14. 10.1016/j.cmet.2011.07.012 21907145

[80] Nijhout HF. Genes on the wing. Science. 1994;265(5168):44–5. 7912450 10.1126/science.7912450

[81] Nijhout HF, Riddiford LM, Shingleton MC, Suzuki Y, Callier V. The developmental control of size in insects. WIREs Developmental Biology. 2013;3:113–34. 10.1002/wdev.124 24902837 PMC4048863

[82] Harbison ST, Chang S, Kamdar KP, Mackay TF. Quantitative genomics of starvation stress resistance in *Drosophila*. Genome Biol. 2005;6(4):R36 10.1186/gb-2005-6-4-r36 15833123 PMC1088964

[83] Moskalev A, Zhikrivetskaya S, Krasnov G, Shaposhnikov M, Proshkina E, Borisoglebsky D, et al A comparison of the transcriptome of *Drosophila melanogaster* in response to entomopathogenic fungus, ionizing radiation, starvation and cold shock. BMC Genomics. 2015;16 Suppl 13:S8. 10.1186/1471-2164-16-S13-S8 PMC4686790 26694630

[84] Schuler AM, Wood PA. Mouse models for disorders of mitochondrial fatty acid beta-oxidation. ILAR J. 2002;43(2):57–65. 11917157 10.1093/ilar.43.2.57

[85] Caldwell PE, Walkiewicz M, Stern M. Ras activity in the *Drosophila* prothoracic gland regulates body size and developmental rate via ecdysone release. Curr Biol. 2005;15(20):1785–95. 10.1016/j.cub.2005.09.011 16182526

[86] Francis VA, Zorzano A, Teleman AA. dDOR is an EcR coactivator that forms a feed-forward loop connecting insulin and ecdysone signaling. Curr Biol. 2010;20(20):1799–808. 10.1016/j.cub.2010.08.055 20888228

[87] Gulia-Nuss M, Robertson AE, Brown MR, Strand MR. Insulin-like peptides and the target of rapamycin pathway coordinately regulate blood digestion and egg maturation in the mosquito *Aedes aegypti*. PLoS One. 2011;6(5):e20401 10.1371/journal.pone.0020401 21647424 PMC3103545

[88] Mutti NS, Dolezal AG, Wolschin F, Mutti JS, Gill KS, Amdam GV. IRS and TOR nutrient-signaling pathways act via juvenile hormone to influence honey bee caste fate. J Exp Biol. 2011;214(23):3977–84. 22071189 10.1242/jeb.061499 PMC3212421

[89] Tatar M, Kopelman A, Epstein D, Tu MP, Yin CM, Garofalo RS. A mutant *Drosophila* insulin receptor homolog that extends life-span and impairs neuroendocrine function. Science. 2001;292(5514):107–10. 10.1126/science.1057987 11292875

[90] Hiruma K, Riddiford LM. Developmental expression of mRNAs for epidermal and fat body proteins and hormonally regulated transcription factors in the tobacco hornworm, *Manduca sexta*. J Insect Physiol. 2010;56(10):1390–5. 10.1016/j.jinsphys.2010.03.029 20361974

[91] World Health Organization. World Malaria Report. Geneva: World Health Organization, 2015.

