# Supplementary material for: Transcriptome Sequencing Reveals Large-Scale Changes in Axenic Aedes aegypti Larvae
Source: PLoS Negl Trop Dis. 2017 Jan 6;11(1):e0005273. doi: 10.1371/journal.pntd.0005273 (PMC5245907; doi:10.1371/journal.pntd.0005273)

Fig S1. Representative images of bacteria in the guts of conventional (CNR), and gnotobiotic (GNT) larvae at 18 h post-hatching showing. Bacteria (B) in the gut of conventional larvae were labeled with a peptidoglycan primary antibody visualized using an Alexa Fluor 488 secondary antibody (green) while *E. coli* in gnotobiotic larvae expressed green fluorescent protein. Domains corresponding to the foregut (FG), gastric caecae (GC), anterior midgut (AM), posterior midgut (PM), Malpighian tubules (MT), hindgut (HG) are indicated at the top of the figure. Note the very similar distribution of bacteria in each treatment (scale bar = 500 μm)

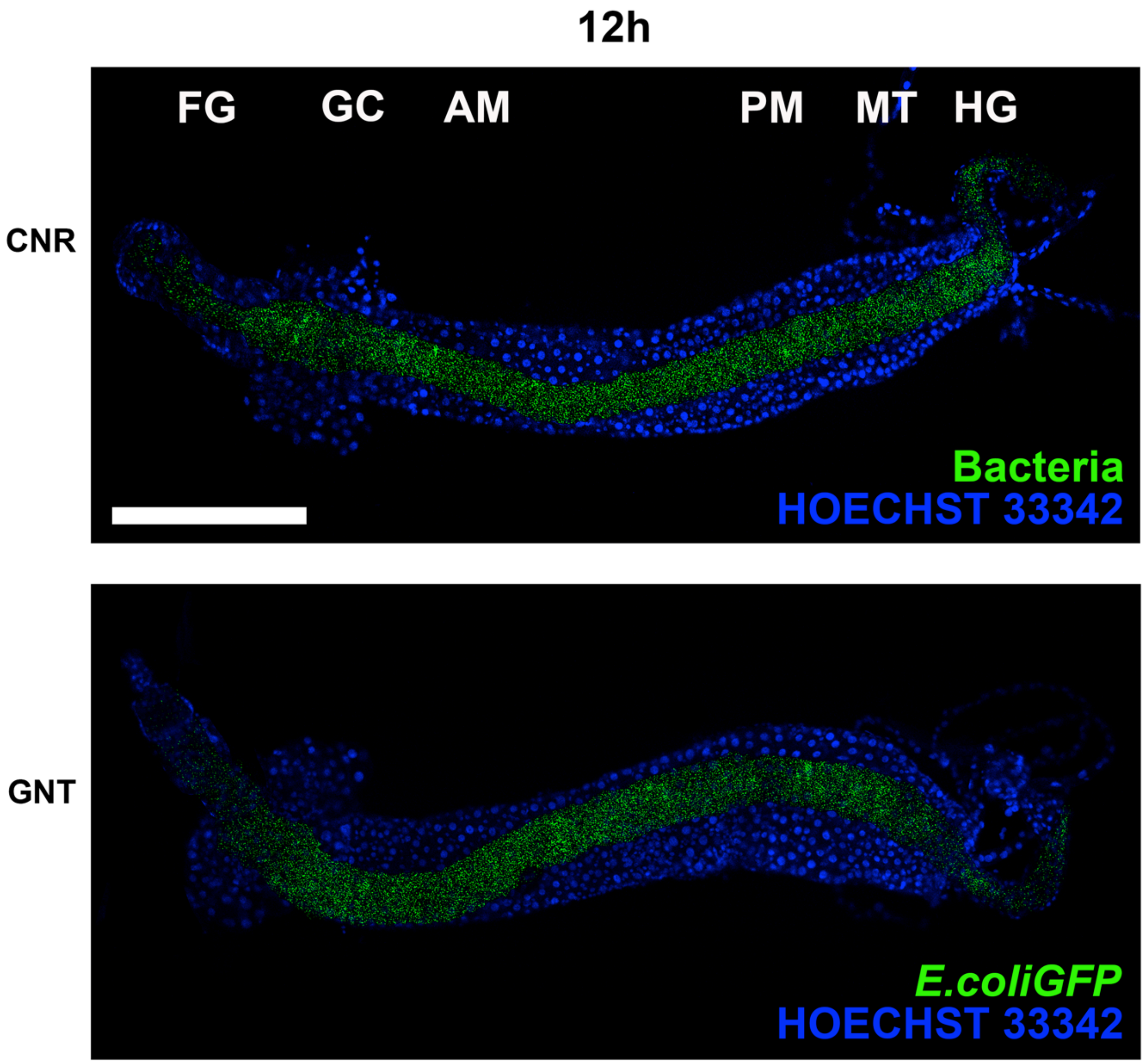

Supplement: S1 Fig — Bacteria (B) in the gut of conventional larvae were labeled with a peptidoglycan primary antibody and visualized using an Alexa Fluor 488 secondary antibody (green) while E. coli in gnotobiotic larvae expressed green fluorescent protein. Domains corresponding to the foregut (FG), gastric caecae (GC), anterior midgut (AM), posterior midgut (PM), Malpighian tubules (MT), and hindgut (HG) are indicated at the top of the figure. Note the very similar distribution of bacteria in each treatment (scale bar = 500 μm). (PDF) [file pntd.0005273.s001.pdf]
