# Supplementary material for: Transcriptome Sequencing Reveals Large-Scale Changes in Axenic Aedes aegypti Larvae
Source: PLoS Negl Trop Dis. 2017 Jan 6;11(1):e0005273. doi: 10.1371/journal.pntd.0005273 (PMC5245907; doi:10.1371/journal.pntd.0005273)
Supplement: S1 Table — (PDF) [file pntd.0005273.s002.pdf]

Table S1: Quality filtering statistics of RNAseq reads

Condition	Tissue	Replicate	Raw reads	Filtered pairs	Total raw reads	Total filtered reads
Axenic	Carcass	1	14.08	5.40	50.04	20.18
		2	21.08	6.55		
		3	14.88	8.23		
	Gut	1	13.39	9.90	38.08	22.90
		2	11.94	5.89		
		3	12.75	7.11		
Gnotobiotic	Carcass	1	15.58	5.03	39.71	15.79
		2	10.72	6.12		
		3	13.41	4.64		
	Gut	1	12.52	5.61	44.25	16.78
		2	16.58	5.06		
		3	15.15	6.11		
Conventional	Carcass	1	166.10	9.82	130.64	7.77
		2	137.22	8.07		
		3	88.61	5.42		
	Gut	1	91.92	5.73	82.90	5.26
		2	71.85	4.40		
		3	84.92	5.65		
**Average**			**45.15**	**6.37**	**64.27**	**14.78**
**Total**			**812.70**	**114.73**		

airs
