# Supplementary material for: Transcriptome Sequencing Reveals Large-Scale Changes in Axenic Aedes aegypti Larvae
Source: PLoS Negl Trop Dis. 2017 Jan 6;11(1):e0005273. doi: 10.1371/journal.pntd.0005273 (PMC5245907; doi:10.1371/journal.pntd.0005273)
Supplement: S2 Table — (PDF) [file pntd.0005273.s003.pdf]

Table S2: Read mapping statistics

Tissue	Treatment	Replicate	Left			Right			Pairs			
			Input	Mapped	Multiple	Input	Mapped	Multiple	Aligned pairs (%)	Multiple alignments	Discordant	Concordant
Carcass	Axenic	1	5.84	3.94	4.53%	5.83	4.00	4.5%	3.69 (63.2%)	4.5%	0.4%	63.0%
		2	8.84	5.93	4.65%	8.84	6.01	4.7%	5.55 (62.8%)	4.6%	0.4%	62.5%
		3	6.43	4.57	4.08%	6.43	4.62	4.1%	4.27 (66.5%)	4.1%	0.4%	66.3%
	Gnotobiotic	1	6.10	4.29	4.06%	6.09	4.35	4.1%	4.01 (65.8%)	4.0%	0.4%	65.6%
		2	4.33	3.09	4.16%	4.33	3.12	4.2%	2.89 (66.9%)	4.2%	0.4%	66.7%
		3	5.64	4.09	4.15%	5.64	4.13	4.2%	3.84 (68.3%)	4.2%	0.3%	68.0%
	Conventional	1	4.84	3.42	8.40%	4.84	3.42	8.4%	3.41 (70.6%)	8.4%	0.0%	70.5%
		2	3.98	2.81	8.60%	3.98	2.81	8.6%	2.81 (70.6%)	8.6%	0.0%	70.6%
		3	2.66	1.89	8.40%	2.66	1.89	8.4%	1.89 (8.3%)	8.3%	0.0%	71.2%
Midgut	Axenic	1	5.51	4.06	5.01%	5.51	4.10	5.0%	3.77 (68.5%)	5.0%	0.4%	68.3%
		2	5.06	3.74	4.48%	5.06	3.78	4.5%	3.49 (69.%)	4.5%	0.4%	68.8%
		3	5.48	4.20	4.68%	5.48	4.24	4.7%	3.93 (71.8%)	4.7%	0.5%	71.5%
	Gnotobiotic	1	5.25	4.00	5.01%	5.25	4.03	5.0%	3.72 (71.%)	5.0%	0.5%	70.7%
		2	6.80	5.13	4.84%	6.80	5.17	4.8%	4.76 (71%)	4.9%	0.5%	69.8%
		3	6.43	4.95	4.95%	6.43	4.99	4.9%	4.62 (72.%)	5.0%	0.5%	71.7%
	Conventional	1	2.81	2.16	8.40%	2.81	2.16	8.4%	2.16 (76.8%)	8.4%	0.0%	76.8%
		2	2.17	1.67	8.40%	2.17	1.67	8.4%	1.66 (76.8%)	8.4%	0.0%	76.8%
		3	2.74	2.05	9.70%	2.74	2.05	9.7%	2.04 (74.6%)	9.7%	0.0%	74.5%
Average			5.1	3.7	5.92%	5.0	3.7	5.9%	3.5 (66.4%)	5.9%	0.3%	69.6%
